# Transcriptomic Analysis Reveals mRNA and Alternative Splicing Events in Ovine Skeletal Muscle Satellite Cells during Proliferation and Differentiation

**DOI:** 10.3390/ani13061076

**Published:** 2023-03-16

**Authors:** Qian Chen, Chang Huang, Yinxiao Su, Qian Zhao, Yabin Pu, Xiaohong He, Lin Jiang, Yuehui Ma, Qianjun Zhao, Shaohui Ye

**Affiliations:** 1 Department of Animal Breeding and Reproduction, College of Animal Science and Technology, Yunnan Agricultural University, Kunming 650201, China; 2 Institute of Animal Science, Chinese Academy of Agricultural Sciences (CAAS), Beijing 100193, China; 3 CAAS-ILRI Joint Laboratory on Livestock and Forage Genetic Resources, Institute of Animal Science, Chinese Academy of Agricultural Sciences, Beijing 100193, China

**Keywords:** ovine skeletal satellite cell, mRNA, alternative splice, transcription factor

## Abstract

**Simple Summary:**

Skeletal muscle satellite cells (SMSCs) serve as the source of myogenic cells and can afford to differentiate into myotubes as well as act as a model for exploring myogenesis in vitro. In this study, the transcriptional profile of ovine skeletal muscle satellite cells was constructed via the RNA-Seq method. A total of 1954 DEGs, 1479 AS, and 253 TFs were detected during the proliferation and differentiation of SMSCs. GO and KEGG analyses showed that the MAPK signaling pathway, PI3K-Akt signaling pathway, Wnt signaling pathway, and Ras signaling pathway were enriched. Together, our study provides novel insights into the transcription regulation of SMSCs during proliferation and differentiation at the transcriptional level, and provides a valuable resource for understanding the molecular mechanism of myogenesis and muscle development.

**Abstract:**

Skeletal muscle satellite cells (SMSCs), which are highly multifunctional muscle-derived stem cells, play an essential role in myogenesis and regeneration. Here, the transcriptional profile of SMSCs during proliferation and differentiation were constructed using the RNA-Seq method. A total of 1954 differentially expressed genes (DEGs) and 1092 differentially alternative splicing genes (DAGs) were identified including 1288 upregulated genes as well as 666 downregulated genes. GO and KEGG analyses showed that the DEGs and DAGs were enriched in the MAPK (mitogen-activated protein kinase) signaling pathway, the PI3K-Akt (phosphatidylinositol-tris-phosphate kinase 3/protein kinase B) signaling pathway, the Wnt signaling pathway, and the Ras signaling pathway. In total, 1479 alternative splice events (AS) were also identified during SMSC proliferation and differentiation. Among them, a unique AS event was the major per-mRNA splicing type, and SE was the predominant splicing pattern. Furthermore, transcription factors with AS were scanned during SMSC differentiation such as myocyte enhancer factor-2C (*MEF2C*) and the nuclear receptor subfamily 4 group A member 2 (*NR4A2*). Our results imply that *MEF2C* and *NR4A2* can interact, and we speculate that *NR4A2* and *MEF2C* might regulate the myogenesis of ovine SMSCs through interaction. Together, our study provides useful information on the transcriptional regulation of SMSCs during proliferation and differentiation at the transcriptional level, and provides a valuable resource for understanding the molecular mechanism of myogenesis and muscle development.

## 1. Introduction

Skeletal muscle is composed of multinucleated and nondividing muscle cells (fibers). Myogenesis is a highly ordered and complex process, whereby myoblasts fuse in a manner finely regulated by various myogenic regulatory factors (MRFs) such as *Myf5*, *MyoD*, *MRF4*, and myogenin [1,2]. MRFs serve as master transcription factors that are upregulated during myogenesis and cause stem cells to differentiate into myogenic lineage cells [1,3,4,5,6]. Skeletal muscle satellite cells are a type of muscle-derived stem cell lying between the myofiber sarcolemma and basal lamina, which are generally quiescent and can be induced to differentiate into adipocytes, osteocytes, and myoblasts in vitro [7,8,9]. Satellite cells serving as the only source of myogenic cells can afford to differentiate into myotubes [10]. It has been shown that damage to muscles or muscle diseases can activate skeletal muscle satellite cells [11,12,13]. The absence of satellite cells severely hampers myogenic differentiation during the initial wave of muscle regeneration [14]. Furthermore, studies have been reported on myoblast proliferation and differentiation study of the transcriptome of many species such as mice [15,16], geese [17], goats [18], and pigs [19], but there are few transcriptome studies involving ovine skeletal muscle satellite cells.

Alternative splicing (AS) is one of the most important contributors of different protein isoforms produced from the same gene, resulting in the high complexity of eukaryotic transcriptomes [20]. The AS events of precursor messenger RNA (pre-mRNA) occur due to the involvement of five small nuclear ribonucleoprotein (snRNP) complexes in the removal of introns [21]. In general, AS events display five patterns: exon skipping (ES), intron retention (IR), mutually exclusive exons (MEE), alternative acceptor site (AAS), and alternative donor site (ADS) [22]. Previous studies have indicated that the AS is associated with the regulation of proliferation [23], differentiation [24,25], and apoptosis [26] of the cell. In addition, a recent report has demonstrated that AS events result in the proliferation and differentiation of myoblasts. A typical example is the gene encoding the myocyte enhancer factor 2 (MEF2) family of transcription factors that form isoforms by extensive alternative splicing. There are four isoforms (*MEF2A*, *MEF2B*, *MEF2C*, and *MEF2D*) in the MEF2 family, which play an important role in cell proliferation and differentiation [27].

In this study, we performed RNA-Seq analysis on ovine skeletal muscle satellite cells at the proliferation and differentiation stages (7 days of differentiation), and screened differentially expressed genes, alternative splice events, and transcription factors associated with muscle development. This study identified potential DEGs and DAGs associated with proliferation and differentiation in SMCSs, and suggests their potential roles in the skeletal muscle development of sheep. These results will provide new insights that are useful for further studies on the molecular mechanism of myogenesis.

## 2. Materials and Methods

### 2.1. Culture and Induced Differentiation of Sheep Muscle Satellite Cells

As previously described, ovine skeletal muscle satellite cells (SMSCs) were isolated from the gastrocnemius muscle of fetal sheep via the 2-step digestion method [9]. In the proliferation stage, the SMSCs were cultured in growth media containing DMEM/F12 (Invitrogen, Carlsbad, CA, USA) with 20% fetal bovine serum (Invitrogen, Carlsbad, CA, USA), 10% horse serum (Invitrogen, Carlsbad, CA, USA), and 1% penicillin-streptomycin (Invitrogen, Carlsbad, CA, USA). In the differentiation stage, the SMSCs were treated with differentiation medium including 2% horse serum upon reaching about 60% confluence. The SMSC samples from the proliferation stage and after 7 days of differentiation were collected for subsequent experiments. All of the samples were kept at −80 °C before RNA extraction.

### 2.2. Immunofluorescent Analysis

The SMSCs were fixed immediately with 4% paraformaldehyde for 1 h at room temperature, then washed with cold PBS (Thermo Fisher, Waltham, MA, USA) three times. Next, the SMSCs were permeabilized with 0.25% Triton-X (Sigma-Aldrich, St. Louis, MO, USA) for 1 h and then blocked with 3% normal goat serum (Bioss, Wuhan, China) for 1 h and stained with the indicated anti-Pax7 (1:100, Bioss, Wuhan, China) and anti-MyHC (1:100, Abmart, Shanghai, China) at 37 °C for 1 h. The secondary antibody (1:200, Abcam, Cambridge, UK) was incubated for 1 h after washing with cold PBS three times. Finally, the slides were repeatedly rinsed with cold PBS and counterstained with DAPI for 15 min. The slides were imaged under a confocal microscope.

### 2.3. Library Construction and Sequencing

The total RNA of six samples was extracted from the SMSCs using the Trizol reagent (Invitrogen) following the manufacturer’s instructions. The concentrations of RNAs were detected by NanoDrop 2000 and the integrity and RNA Integrity Number (RIN) of RNA were assessed by RNase-free agarose gel electrophoresis and an Agilent 2100 Bioanalyzer, respectively. After the rRNA-depleted RNA was fragmented, the RNA-Seq library was constructed using enriched poly (A)-tailed of messenger RNA (mRNA) by magnetic beads with Oligo (dT) (Invitrogen). The enriched mRNA was broken into the fragments and reversely transcribed into first-strand cDNAs. Second-strand cDNAs were obtained using DNA polymerase I (Thermo Fisher) and RNase H (Thermo Fisher). The fragmented mRNA was purified and PCR amplification was performed. Finally, six libraries were sequenced on the lllumina NovaSeq 6000 platform with the PE150 model.

### 2.4. Transcripts Assembly

First, the adaptor, contaminated reads, ploy-N reads (with quality less than 3), low-quality reads, the reads with a length less than 50 bp, and duplicated reads were removed from the raw data using the fastp software. Then, the rRNA was removed from the clean data by mapping the silva database using bowtie2 software [28], and clean data with removed rRNA were mapped to the ovine reference genome (Oar_rambouillet_v1.0) by hisat2 software [29]. The fragments per kilobase of transcript per million mapped reads (FPKM) value of the six samples were calculated to determine the gene expression level using StringTie software [30].

### 2.5. RT-qPCR Validation

The total RNA of the six samples was reverse transcribed to cDNA using the HiScript^®^ III All-in-one RT SuperMix Perfect for qPCR (Vazyme, Nanjing, China) following the manufacturer’s instructions. Then, qPCR was performed using the Taq Pro Universal SYBR qPCR Master Mix (Vazyme, Nanjing, China) according to the manufacturer’s instructions. Six genes were randomly selected for the validation of the RNA-Seq results including *THBS2*, *SACS*, *MCM4*, *COL1A1*, *ACTC1*, and *MFAP4*; the *GAPHD* and *ꞵ-Actin* genes were used as the internal reference gene and then calculated using the 2^−∆∆Ct^ method. Each qPCR reaction was performed in a 20 μL reaction mixture that included 2 μL template cDNA, 0.8 μL of 10 uM forward and reverse primers, 10 μL Taq Pro Universal SYBR qPCR Master Mix, and 6 μL RNase-free water. The qPCR amplification contained an initial denaturation step (95 °C for 10 s) and 40 cycle stages of 10 s at 95 °C and 30 s at 60 °C. All primers used in the RT-qPCR are shown in Table 1.

### 2.6. Alternative Splicing Gene Analysis

Alternative splice events were identified using the rMATS software [31]. In brief, five major AS events were identified in the aligned BAM file and merged with the reference GTF file including skipped exon (SE), alternative 5′ splice site (A5SS), alternative 3′ splice site (A3SS), mutually exclusive exons (MXE), and retained intron (RI). The differentially expressed genes with alternative splice (DAGs) were screened into two groups with a false discovery rate (FDR) of <0.01.

### 2.7. Differential Expression and Functional Enrichment Analysis

The differentially expressed mRNAs were identified according to a |log2 (Fold Change)| > 2 and *q*-value < 0.01 using the R package in edgeR. To explore the functions of differentially expressed mRNAs, we performed Gene Ontology (GO, https://biit.cs.ut.ee/gprofiler/gost accessed on 8 January 2023) and Kyoto Encyclopedia of Genes and Genomes (KEGG, http://kobas.cbi.pku.edu.cn/genelist accessed on 8 January 2023) analyses. GO terms and KEGG pathways with a *p*-value (*p* < 0.05) were considered significantly enriched terms and pathways.

### 2.8. PPI Analysis

The top 50 most upregulated genes and downregulated genes were calculated using the STRING database (https://cn.string-db.org accessed on 26 February 2023), and the protein−protein interaction (PPI) network was inferred and visualized using Cytoscape (v3.9.1). The node colors represent the corresponding interaction genes, and the node sizes represent the upregulated and downregulated DEGs.

## 3. Results

### 3.1. Identification and Overview of Transcriptome Sequencing of Ovine Skeletal Muscle Satellite Cell at Different Stages

**Figure 1:**
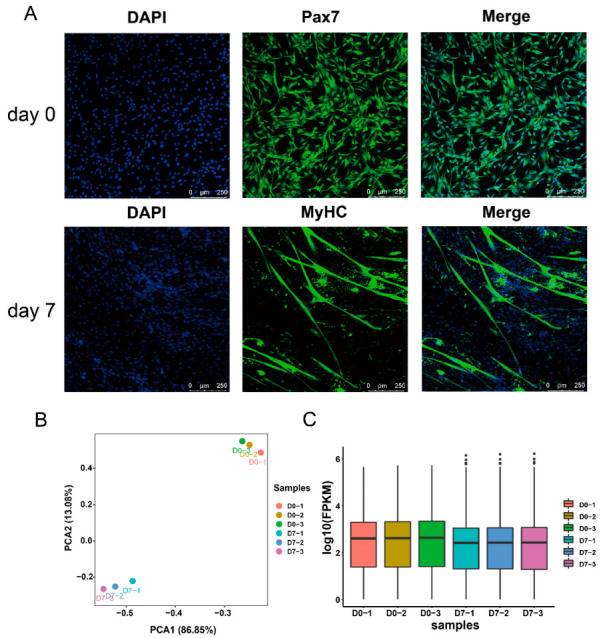
Ovine skeletal muscle satellite cell isolation and gene expression levels. (**A**) Immunofluorescent staining results at the proliferation and differentiation stages; (**B**) principal component analysis of six samples; (**C**) the expression level of mRNAs; FPKM represents the fragments per kilobase of transcript per million mapped reads.

Ovine skeletal muscle satellite cells (SMSCs) were isolated from fetal sheep skeletal muscles by the collagenase digestion method. As expected, a 90% proliferation confluence of ovine SMSCs was observed by day 2 in the in vitro culture (Figure 1A). When the cell density reached about 70%, they were induced into differentiation by a 2% horse serum medium, which resulted in myotubes (Figure 1A). To further characterize the cell proliferation and differentiation, we performed an immunofluorescent assay on SMSCs during the proliferation and differentiation phases. The results showed that Pax7 was positive in the satellite cells at the proliferation stage and MyHC was highly expressed at the differentiation stage (Figure 1A), which indicates that the ovine SMSCs were successfully induced into differentiation.

Next, to investigate the gene expression profiles in the proliferation and differentiation stages of SMSCs, transcriptome sequencing analysis was performed. More than 69.57 million clean reads were acquired for the sequencing analysis, and above 92% of the clean reads were successfully aligned on the sheep reference genome for each sample. The Q20 and Q30 values of samples were higher than 90%, and the GC content ranged from 47% to 48% (Table 2). Additionally, the principal component analysis (PCA) demonstrated that the SMSC samples in the proliferation stage were distinct from those in the differentiation stage (Figure 1B). The box diagram shows that higher gene expression levels were observed in the proliferation stage than in the differentiation stage (Figure 1C).

### 3.2. Transcriptional Profiling of mRNA Expression in SMSCs at Proliferation and Differentiation Stages

**Figure 2:**
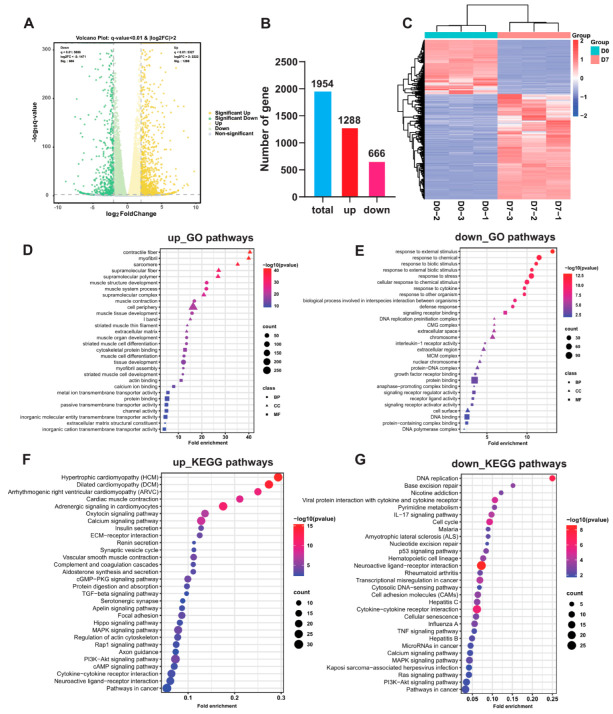
The expressions and functional analyses of the DEGs in SMSCs at the proliferation and differentiation stages. The schemes follow the same formatting. (**A**) The volcano plot of DEGs between groups D0 and D7 groups |Log2 fold change| > 2, *q* < 0.01; the *q* value was adjusted to the *p* value by multiple hypothesis testing. (**B**) The number of DEGs. (**C**) Heat maps showing DEGs were expressed between the D0 and D7 groups, red denotes a high level of expression, whereas blue denotes a low level of expression. (**D**) GO analysis showing the top 30 pathways of the upregulated DEGs. (**E**) GO analysis showing the top 30 pathways of the downregulated DEGs. (**F**) KEGG analysis showing the top 30 pathways of upregulated DEGs. (**G**) KEGG analysis showing the top 30 pathways of downregulated DEGs (*p* < 0.05). Note: The size of the circle indicates the number of annotated differentially expressed genes, and the color represents the −log *p* value.

In total, 1954 differently expressed mRNAs (DEGs) were identified in SMSCs at the proliferation and differentiation stages including 1288 upregulated and 666 downregulated DEGs (Figure 2A,B). The clustered heatmap shows that the DEGs were clearly distinct between the differentiation and proliferation stages (Figure 2C), indicating that dynamic changes in the genes occurred during the proliferation and differentiation periods. The results showed that the DEGs weakly expressed in the cell proliferation stage were highly expressed to regulate cell differentiation such as *MYH1*, *MYBPC1*, *ACTC1*, *MEF2C*, and *IGFBP5*. Downregulated DEGs were highly expressed during proliferation and expressed at low levels in differentiation such as *AREG* and *IL11* (Supplementary Table S1). Therefore, we speculated that the majority of upregulated DEGs might promote ovine skeletal muscle satellite cell differentiation.

Next, the GO and KEGG pathway enrichment analyses were performed on the DEGs. Regarding the upregulated DEGs, the GO analysis showed that the DEGs were mainly enriched in the biological processes related to muscle development such as muscle system processes, cell periphery, muscle tissue development, actin binding, and muscle cell differentiation (Figure 2D). The KEGG pathways of DEGs mainly included cardiac muscle contraction, the TGF-beta signaling pathway, MAPK signaling pathway, Hippo signaling pathway, Rap1 signaling pathway, PI3K-Akt signaling pathway, and cAMP signaling pathway (Figure 2F, Supplementary Table S2). For the downregulated DEGs, the GO results show that DEGs were enriched in protein binding and DNA binding (Figure 2E). The KEGG pathways of DEGs included cell cycle, the MAPK signaling pathway, PI3K-Akt signaling pathway, and the Ras signaling pathway (Figure 2G, Supplementary Table S3).

### 3.3. Protein–Protein Interaction (PPI) Network of Differentially Expressed Genes in SMSCs at Proliferation and Differentiation Stages

**Figure 3:**
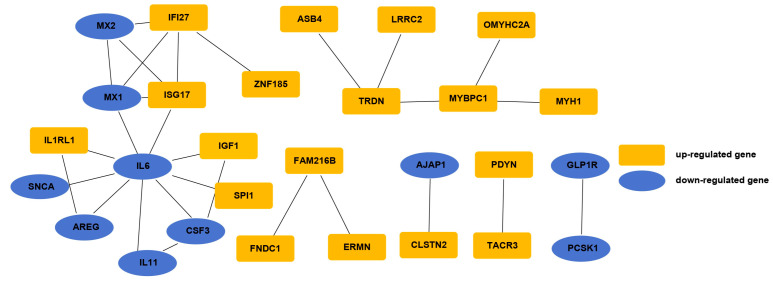
Top 50 most upregulated and downregulated DEGs from the protein–protein interaction (PPI) network. Blue nodes represent the downregulated DEGs, and the yellow nodes represent the upregulated DEGs.

As shown in Figure 3, the PPI network based on the top 50 most upregulated DEGs and the top 50 most downregulated DEGs consisted of 28 nodes and 29 interaction pairs. The PPI network included eight interaction pairs for downregulated DEGs, 10 interaction pairs for upregulated DEGs, and 11 interaction pairs between the upregulated and downregulated DEGs. The results showed that IL6 correlated with nine other genes including IGF1, IL11, and AREG, and MYBPC1 correlated with three other genes including MYH1, which is involved in the proliferation and differentiation of SMSCs.

### 3.4. Validation of the Differentially Expressed Genes

**Figure 4:**
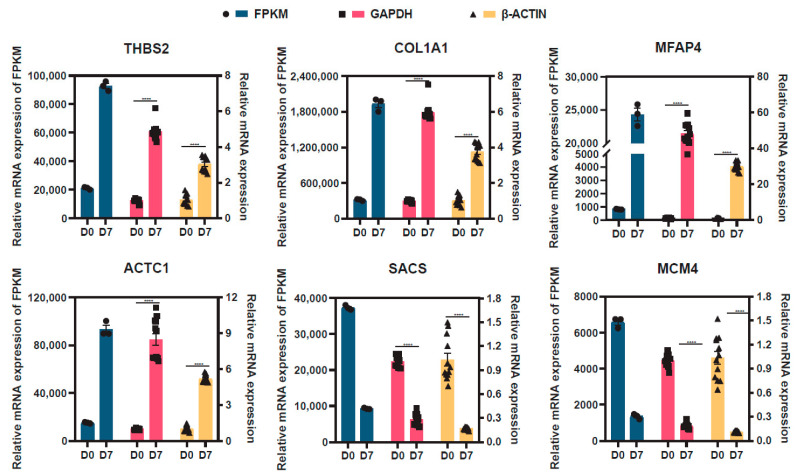
RT-qPCR validation of six differentially expressed genes. The left *Y*-axis displays the FPKM derived from the RNA-Seq, while the data from RT-qPCR are shown on the *Y*-axis on the right. Note: The data are represented as the means ± SED; blue represents the FPKM value of RNA-Seq, pink represents the RT-qPCR with *GAPDH* as the internal reference gene, and yellow represents the RT-qPCR with *ꞵ-ACTIN* as the internal reference gene; **** means *p* < 0.001.

In order to determine the accuracy and validity of the RNA-Seq results, six differentially expressed genes (four upregulated and two downregulated genes) including *THBS2*, *COL1A1*, *MFAP4*, *ACTC1*, *SACS*, and *MCM4* were detected using the RT-qPCR method. The *GAPDH* and *ꞵ-ACTIN* genes were used as the internal reference gene. Consistent with the RNA-Seq results, *THBS2*, *COL1A1*, *MFAP4*, and *ACTC1* were upregulated at the differentiation stage, and *SACS* and *MCM4* were downregulated at the differentiation stage (Figure 4).

### 3.5. Alternative Splicing Analysis of mRNA in SMSCs during Proliferation and Differentiation

**Figure 5:**
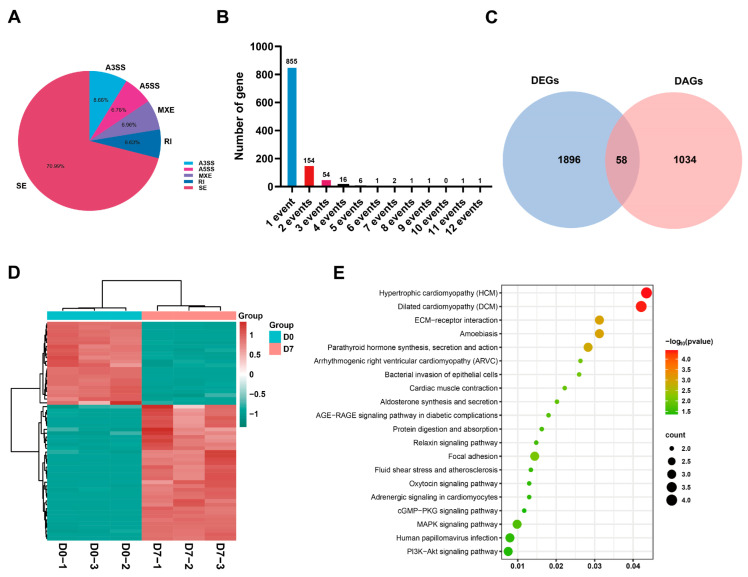
Differential splicing analysis of DEGs between the proliferation stage and differentiation stage of SMSCs. (**A**) Distribution of five AS events. SE: skipped exon, A3SS: alternative 3′ splice site, A5SS: alternative 5′ splice site, RI: retained intron, MXE: mutually exclusive exons. (**B**) Differential splicing events between proliferation stage and differentiation stage; (**C**) Venn diagram depicting DEGs and DAGs. (**D**) The heatmap of 58 overlapped genes, where red denotes a high level of expression, whereas green denotes a low level of expression; (**E**) KEGG pathways of the overlapping DEGs and DAGs, where the size of the circle indicates the number of annotated differentially expressed genes, and the color represents the −log *p* value.

Most of the multi-exon genes underwent alternative splicing, generating complex and diverse transcripts [32,33]. It has been extensively demonstrated that alternative splicing events in myogenesis are important to proper skeletal muscle development [34,35]. To evaluate the extent and significance of AS in myogenesis, we performed alternative splicing analysis on SMSCs during proliferation and differentiation. A total of 1479 differentially expressed AS events were identified in 1092 genes including 1050 skipped exons (SE), 98 remained introns (RI), 128 alternative 3′ splice sites (A3SS), 100 alternative 5′ splice sites (A5SS), and 103 (MXE) mutually exclusive exons, of which SE accounted for 70.99% of these AS (Figure 5A). Furthermore, genes showing a unique AS event accounted for 78.3% of the total, making this major type of AS event during myogenesis differentiation, followed by 154, 54, 16, and six genes displaying two, three, four, and five AS events, respectively (Figure 5B). Among these, 58 genes were significantly differentially expressed in the genes with alternative splice sites (Figure 5C,D), of which several genes have been reported to be related to myogenesis or muscle development such as *MEF2C*, *MYOM3*, *MYO5B*, *CEMIP*, and *ITGB6* (Supplementary Table S4). To further understand the function of these differentially expressed genes with alternative splice sites arising in SMSCs during proliferation and differentiation, we performed GO and KEGG analyses of 58 overlapping genes. KEGG analysis showed that these overlapping genes were most significantly enriched in cardiac muscle contraction, the MAPK signing pathway, PI3K-Akt signing pathway, and the processes of hormones (Figure 5E). Collectively, these results revealed that the unique AS event was the major per-mRNA splicing type and SE was the predominant splicing pattern, which may be involved in regulating the proliferation and differentiation of SMSCs.

### 3.6. Transcription Factors Identified during SMSCs Proliferation and Differentiation Stages

**Figure 6:**
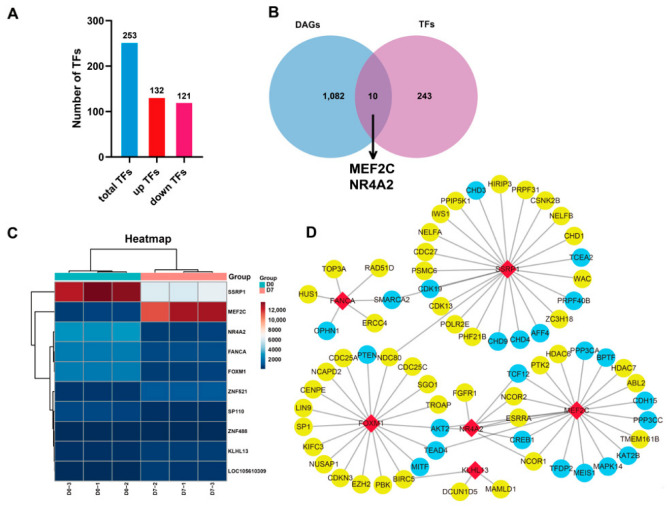
Transcription factor analysis of DEGs in SMSCs between the myoblasts and myotubes. (**A**) The number of TFs in upregulated and downregulated TFs (|log2 Fold Change| > 1). (**B**) Venn diagram of DAGs and TFs. (**C**) Heatmap of the nine transcription factors. (**D**) The protein–protein analysis of DAGs and TFs with differentially expressed genes split into two groups. Red, transcription factor; blue, upregulated genes; yellow, downregulated genes.

As transcription factors play important roles in the regulation of gene transcription, we identified those that were differentially expressed transcription factors. A total of 253 DEGs were defined as transcription factors (TFs), comprising 132 up-regulated and 121 down-regulated TFs in myoblasts and myotubes (Figure 6A). In addition, we found 10 TFs (including 5 up-regulated and 5 down-regulated) with alternative splice sites, of which *SSRP1* (structure-specific recognition protein 1) and *MEF2C* (Myocyte enhancer factor 2C) were most significantly differentially expressed (Figure 6B,C). Among the TFs, *MEF2C* and *NR4A2* (nuclear receptor subfamily 4 group A member 2) belonged to significantly differentially expressed genes showing alternative splicing. Further analysis of the PPI network of overlapping DAGs and TFs genes analysis demonstrated that transcription factors interact with DAGs. Among these, *SSRP1*, *FOXM1* (Forkhead box protein M1) and *MEF2C* can be identified as hub genes (Figure 6D).

## 4. Discussion

Myogenesis is a complex biological process that involves multiple gene regulation mechanisms. Transcriptomic analyses of muscles across multiple developmental stages and myoblasts during proliferation and differentiation have also been reported in different species [18,36]. However, there have been a few reports addressing the regulation mechanism at play in ovine skeletal muscle satellite cells. In this study, we constructed the expression profiles of SMSCs in the proliferation and differentiation stages using the RNA-Seq method. The differentially expressed genes (DEGs), alternative splices (AS), and transcription factors (TFs) involved in ovine skeletal muscle satellite cell proliferation and differentiation were identified. A total of 1954 DEGs, 1479 AS, and 253 TFs were detected during the proliferation and differentiation of SMSCs. We found that the expression levels of all genes during proliferation were higher than those during the differentiation of SMSCs, which is consistent with the results of previous studies [17].

Compared with the proliferation stage, we found 1288 upregulated mRNAs and 666 downregulated mRNAs in SMSCs during the differentiation stage including *MYH1*, *MYBPC1*, *ACTC1*, *THBS2*, *COL1A1*, *IL11*, *AREG*, and *IL6*. Consistent with previous studies, our results showed that these mRNAs were enriched in multiple signaling pathways that are relevant to muscle development including the Hippo signaling pathway [37,38], MAPK signaling pathway [39], PI3K-Akt signaling pathway [40], and Ras signaling pathway [41]. Among these, we also observed two KEGG pathways, the MAPK signaling pathway and PI3K-Akt signaling pathway, as the most commonly enriched pathways in the upregulated and downregulated genes. It has been reported that the MAPK pathway is composed of a series that operates in the protein kinase cascade, which plays an important roles in the regulation of cell proliferation [42] and differentiation [43]. Furthermore, these DEGs are closely related to the proliferation and differentiation of muscle cells such as *ACTC1*. Several studies have demonstrated that these genes may profoundly contribute to the expansion and differentiation of SMSCs. For instance, *ACTC1* (actin alpha cardiac muscle 1) is involved in the differentiation of myoblasts, and *ACTC1* deficiency led to severe structural and functional perturbations in the heart [44,45]. The knockdown of *COL1A1* inhibits the proliferation of bovine skeletal muscle satellite cells [46]. Therefore, these DEGs are indispensable to the proliferation and differentiation of SMSCs and to muscle development.

In our study, protein–protein interaction (PPI) network analyses of the top 50 most upregulated and downregulated genes showed that DEGs played a significant role in the proliferation and differentiation of SMSCs. For example, interleukin (IL)6 and *MYBPC1* (slow skeletal muscle myosin-binding protein-C) have been found to be significantly associated with the proliferation and differentiation of SMSCs. Although IL6 is principally defined as a proinflammatory cytokine, it also potentially triggers and controls the distinct activities of satellite cells throughout the myogenic process [47]. The knockout of IL6 in differentiated C2C12 myoblasts can impair the myotube fusion [48]. Among them, *IGF1*, *AREG*, and *IL11* were predicted to interact with IL6, which is reportedly involved in satellite cell proliferation and differentiation [49,50,51]. With regard to the upregulated genes, *MYBPC1* has been reported to play an important role in normal muscle growth and development processes [52,53,54]. For example, *MYBPC1* mutations exert negative effects on muscle function, resulting in an embryo with mild curvature and impaired mobility [55].

Moreover, this study also analyzed the AS events at play during ovine myoblast proliferation and differentiation based on transcriptomic data. In order to more effectively explore the AS events involved in regulation during myoblast differentiation, we highlighted the overlapping genes between DEGs and DAGs such as *MEF2C* [56]. Two main isoforms of *MEF2C* have been found. In zebrafish, *MEF2Cb* is a *MEF2C* paralogue that predominates during somitogenesis, and the overexpression of *MEF2Cb* leads to the ectopic expression of both cardiac and skeletal muscle related genes [57,58]. The *MEF2C* isoform is reported to induce cell cycle reentry and the development of heart failure in cardiomyocytes [59]. Additionally, these overlapping genes are mainly enriched in the MAPK signaling pathway. Previous studies have demonstrated that some pathways can indirectly impact the biological functions of cells though affecting splicing factors such as the MAPK signaling pathway [60]. For example, MEK1b and ERK1c are the alternatively spliced isoforms of MAPKKs and MAPKs, and MEK1b and ERK1c can form an independent signaling pathway to regulate cell fate [60].

In the development of multicellular organisms, transcription factors determine the fate of individual cells. For the PPI network with the overlap in DAGs and transcription factor, we identified *MEF2C*, *SSRP1*, *NR4A2*, *FOXM1*, *FANCA*, and *KLHL13* as hub genes in networks, which may be related to myogenesis. Previous studies have shown that hub genes are essential for regulation of the cell cycle [61], skeletal muscle differentiation, and growth [62]. For example, the loss of *FOXM1* contributes to cell communication and non-autonomous satellite cell activation in zebrafish skeletal muscle [61]. The interaction of *SSRP1* with SRF dramatically increases the DNA binding activity of SRF, and *SSRP1* interacts with myogenin and promotes myoblast differentiation-specific muscle gene expression [63,64]. Additionally, we also found that *MEF2C* and *NR4A2* were overlapped in DEGs, DAGs, and TFs. Among these, *MEF2C*, a member of the MEF2 family, exhibits a strong interaction and co-expression pattern with other genes, and it can promote myogenic differentiation [65,66]. *NR4A2* (also known as *NURR1*) is upregulated in response to muscle exercise. It was been reported that *NURR1* binds to a site on the MEF2, and *Nurr1* activation controls systemic energy homeostasis in skeletal muscle [67]. Our results showed an interaction between *MEF2C* and *NR4A2*, indicating that there might be a *NR4A2* binding sites on the *MEF2C* gene, which is involved in myogenesis regulation. However, the potential functions and molecular mechanisms of *MEF2C* and *NR4A2* should be investigated in future studies.

## 5. Conclusions

In conclusion, our study provides the expression profiles of the mRNA, alternative splice, and transcription factors of the ovine skeletal muscle satellite cells during proliferation and differentiation. Moreover, we elaborated the roles played by many important gene such as *MEF2C*, *ACTC1*, *FOXM1*, *SSRP1*, *IL6*, *IL11*, and *NR4A2* in muscle growth or the proliferation and differentiation of satellite cells, and we speculated that *NR4A2* and *MEF2C* might regulate the myogenesis of ovine SMSCs through interaction. In future, these genes should be assessed to explore the function and regulatory mechanisms of myogenic differentiation in vivo and in vitro. The present study helps to improves our understanding of the differences in gene expression during myoblast proliferation and differentiation, and provides insights into muscle development, which can offer guidance to meat production in the future.

## Figures and Tables

**Table 1:** Primer sequence for real-time quantitative PCR.

Genes	Forward Primer Sequence (5′→3′)	Reverse Primer Sequence (5′→3′)
*SACS*	GGCCTGGCAGAGAGTTGATT	CACACGTCTGCCCTTCTTCT
*MCM4*	ATCTGCTGCATCGACGAGTT	TGACAGATGATCCCAGCCTTG
*COL1A1*	ACGTGATCTGCGACGAACTT	GGTCCGTGGTTGATTCCTGG
*ACTC1*	TGGATCTAGCTGGTCGGGAT	GATGACTTGGCCATCAGGCA
*MFAP4*	CGGGAAGTGGACGGTTTTC	AAGCCTGACACGTAGAGGGT
*THBS2*	CTGGCATCGCTGTTGGTTTC	AGCCAGCATAGTCATCGTCG
*β-ACTIN*	CCAACCGTGAGAAGATGACC	CCAGAGGCGTACAGGGACAG
*GAPDH*	TGCCATCAATGACCCCTTCA	ATGACGAGCTTCCCGTTCTC

**Table 2:** Summary of the RNA-Seq data for each sample.

Samples	Clean Reads	Total Mapped	Clean Q20	Clean Q30	Clean GC
D0-1	89,082,288	93.66%	96.75%	92.16%	48.00%
D0-2	92,925,334	93.37%	96.45%	91.37%	49.18%
D0-3	102,897,642	93.57%	96.62%	91.84%	48.91%
D7-1	69,573,942	92.48%	96.66%	91.82%	46.89%
D7-2	70,032,866	92.74%	96.66%	91.83%	47.70%
D7-3	72,196,834	93.14%	96.80%	92.20%	48.08%

## Data Availability

The RNA-Seq data generated and analyzed in this study are available in the NCBI Sequence Read Archive database: PRJNA924666.

## Supplementary Materials

The following supporting information can be downloaded at: https://www.mdpi.com/article/10.3390/ani13061076/s1, Supplementary Table S1: The q value and fold changes of differentially expressed genes of the proliferation and differentiation stage of sheep skeletal muscle satellite cell; Supplementary Table S2: KEGG enrichment annotation for the up-regulated differentially expressed genes in the proliferation and differentiation stage of ovine skeletal muscle satellite cells; Supplementary Table S3: KEGG enrichment annotation for the down-regulated differentially expressed genes in the proliferation and differentiation stage of ovine skeletal muscle satellite cells; Supplementary Table S4: The q value and fold changes of 58 overlapping genes of differentially expressed genes and differentially alternative splicing genes in the proliferation and differentiation of ovine skeletal muscle satellite cells.
